# Modulating the p66shc Signaling Pathway with Protocatechuic Acid Protects the Intestine from Ischemia-Reperfusion Injury and Alleviates Secondary Liver Damage

**DOI:** 10.1155/2014/387640

**Published:** 2014-03-16

**Authors:** Lingfei Ma, Guangzhi Wang, Zhao Chen, Zhenlu Li, Jihong Yao, Haidong Zhao, Shu Wang, Zhenhai Ma, Hong Chang, Xiaofeng Tian

**Affiliations:** ^1^Department of General Surgery, The Second Hospital of Dalian Medical University, Dalian 116023, China; ^2^Department of Pharmacology, Dalian Medical University, Dalian 116044, China

## Abstract

Intestinal ischemia-reperfusion (I/R) injury is a serious clinical pathophysiological process that may result in acute local intestine and remote liver injury. Protocatechuic acid (PCA), which has been widely studied as a polyphenolic compound, induces expression of antioxidative genes that combat oxidative stress and cell apoptosis. In this study, we investigated the effect of PCA pretreatment for protecting intestinal I/R-induced local intestine and remote liver injury in mice. Intestinal I/R was established by superior mesenteric artery occlusion for 45 min followed by reperfusion for 90 min. After the reperfusion period, PCA pretreatment markedly alleviated intestine and liver injury induced by intestinal I/R as indicated by histological alterations, decreases in serological damage parameters and nuclear factor-kappa B and phospho-foxo3a protein expression levels, and increases in glutathione, glutathione peroxidase, manganese superoxide dismutase protein expression, and Bcl-xL protein expression in the intestine and liver. These parameters were accompanied by PCA-induced adaptor protein p66shc suppression. These results suggest that PCA has a significant protective effect in the intestine and liver following injury induced by intestinal I/R. The protective effect of PCA may be attributed to the suppression of p66shc and the regulation of p66shc-related antioxidative and antiapoptotic factors.

## 1. Introduction

Intestinal ischemia-reperfusion (I/R) injury is a complex surgical and pathophysiological process which is secondary to many clinical conditions, such as shock, small bowel transplantation, disseminated intravascular coagulation, and sepsis [1–4]. Intestinal I/R injury is an extremely critical condition since restoring the blood supply after mesenteric ischemia results in desquamation of the intestinal epithelium, bacteria translocation, and systemic inflammatory response [5, 6]. Patients of such processes often suffer from fatal conditions, including systemic inflammatory response syndrome (SIRS) and multiple organ dysfunction syndrome (MODS) [7]. MODS is characterized as progressive physiologic dysfunction of two or more organ systems. The organs that most commonly show potential dysfunction include the local intestine [8], liver [9], lungs [10], and kidneys [8]. Of these distant organs, the liver is the first distant organ involved after intestinal I/R, presumably because the vasculature of the liver is coupled in series with intestinal circulation [9, 11–13]. Mechanism studies revealed that the magnitude of intestinal I/R-induced liver injury depends on inflammatory infiltration, reactive oxidant species (ROS) accumulation, and the response of apoptosis [9, 12, 14]. A series of studies has suggested that systemic inflammatory response, oxidative stress, and cell apoptosis play important roles during the development of intestinal I/R injury and subsequent liver damage. Probable mechanisms involved in this process include glutathione (GSH) depletion, nuclear factor-kappa B (NF-*κ*B) pathway activation, and suppression of Nf-E2-related factor 2 (Nrf2) nuclear translocation [14–16]. However, detailed mechanisms of intestinal I/R remain obscure. Recent studies showed that genetic ablation of the adaptor protein p66shc (p66shc) in the mouse reduced ROS production and consequently prolonged the life span by 30% [17], while deletion of p66shc protected mice from I/R-induced brain injury through blunted production of free radicals [18]. However, it is unknown whether p66shc is involved in intestinal I/R injury and subsequent liver dysfunction. Therefore, we examined the redox-related enzyme p66shc and hypothesized that targeted suppression of p66shc would protect against damage during intestinal I/R.

p66shc is one of three isoforms derived from alternative splicing of the Shc locus. It is ubiquitously expressed in mammals and functions as a redox enzyme implicated in mitochondrial ROS generation [19]. The other two isoforms of this family, p46shc and p52shc, have been described as adaptor proteins that can alter mitogen-activated protein kinase and Fos activation upon stimulation with epidermal growth factor and platelet-derived growth factor [20]. p66shc functionally differentiates from these isoforms. Due to its unique NH_2_-terminal region, it has a serine 36 residue that can be phosphorylated, allowing p66shc to be transferred from the cytosol to the mitochondria. p66shc translocation can lead to blunting of Ca^2+^ responses and opening of the mitochondrial permeability transition pore, and thus inducing intracellular ROS overgeneration and cell apoptosis. Evidence is increasingly accumulating to show that p66shc may play a critical role in ROS production and cell apoptosis [17, 21]. p66shc-null mice, unlike wild-type mice, were shown to be protected against atherogenesis, high glucose-induced renal injury, and aging [17, 22, 23]. Additionally, knockdown of p66shc expression or phospho-p66shc suppression ameliorated cardiac ischemia-reperfusion induced oxidative stress injury* in vivo* or improved mouse kidney proximal tubule cells surviving during H_2_O_2_-induced oxidative stress injury* in vitro *[24, 25].

Protocatechuic acid (PCA), which has been widely studied as a polyphenolic compound, is derived from green tea, fruits, and various Chinese herbal medicines such as* Salvia miltiorrhiza* [26]. Recent studies have shown that PCA induced high expression of antioxidative genes and played a significant role in fighting against oxidative stress injury and cell apoptosis [26, 27]. PCA pretreatment can prevent cells from oxidative stress injury by relieving GSH depletion and enhancing the activity of glutathione peroxidase (GSH-PX) and glutathione reductase, which show marked antioxidative activity [27]. Additionally, studies showed that PCA pretreatment significantly attenuated PC12 cell apoptosis by limiting caspase-3 activity and inducing Bcl-2 expression [28]. Notably, a recent study indicated that PCA pretreatment can downregulate p66shc expression and protect against cell apoptosis induced by lipid peroxidation in Caco-2 cells [27]. However, there was no evidence that PCA pretreatment protects against acute intestinal stress injury and liver dysfunction induced by intestinal I/R.

Therefore, in this study, we used an intestinal I/R model in mice to examine the hypotheses that (1) p66shc-dependent oxidative stress and apoptosis mechanisms are involved in local organ damage and remote liver injury induced by intestinal I/R; (2) p66shc suppression by PCA pretreatment may protect against intestinal injury and liver dysfunction induced by intestinal I/R; (3) the protective effect of PCA pretreatment may be related to p66shc-related foxo3a and Bcl-xL signaling pathways.

## 2. Materials and Methods

### 2.1. Experimental Design

The study was performed in adult male ICR mice (18–22 g) (Animal Centre of Dalian Medical University, Dalian, China). The mice were housed at a temperature of 22 ± 2°C, kept on a 12:12-h photoperiod, and were all provided with food and water* ad libitum*. Procedures were conducted in accordance with the institutional guidelines for the care and use of laboratory animals and the study was approved by the Institutional Animal Care Committee of Dalian Medical University (Dalian, China).

### 2.2. Intestinal I/R Model and Experimental Procedures

The mouse intestinal I/R model was established according to previously standardized methods [29]. Briefly, after general anesthesia, a midline laparotomy was performed, and the superior mesenteric artery (SMA) was isolated gently at its origin and occluded with an atraumatic microvascular clamp for 45 min and then followed by reperfusion for 90 min. The occlusion was confirmed by complete pulse cessation and the intestines became pale; then the reperfusion was confirmed by the return of pulsatile flow to the mesenteric artery and its branches. Sham animals underwent the same protocol but the SMA was not occluded. Fifty mice were divided randomly into five groups (*n* = 10): (i) a sham group which underwent sham surgery; (ii) a I/R group which underwent intestinal I/R model as previously mentioned; (iii) a sham + PCA high dose (H) group which underwent sham surgery and pretreated with PCA (J&K Chemical, Germany) at a dose of 80 mg/kg intraperitoneally (i.p.) for 3 consecutive days; (iv) a I/R + PCA low dose (L) group which underwent intestinal I/R model as previously mentioned; PCA was pretreated at a dose of 40 mg/kg i.p. for 3 consecutive days; (v) a I/R + PCA high dose (H) group which underwent intestinal I/R model as previously mentioned and received PCA pretreatment at a dose of 80 mg/kg i.p. for 3 consecutive days. The dose and route of administration were determined according to a previous study [30]. PCA was dissolved in sterile normal saline before administration and the mice in the sham and I/R groups were treated with an equal volume of sterile normal saline for parallel. Blood and tissue for analysis were obtained by placing the mice under anesthesia and reopening the abdominal midline incision. After being sacrificed by abdominal aortic blood drawing, intestine and liver tissue samples were procured from a standard location in the terminal ileum and left lobe of liver. Blood and tissue samples were collected for future analysis.

### 2.3. Intestine and Liver Morphologic Evaluation

For histopathological analysis, the intestine and liver specimens were instantly fixed in 10% buffered formalin phosphate and embedded in paraffin, sectioned transversely (4 *μ*m), and then hematoxylin eosin (H&E) stained. The mucosal injury score was graded on a six-tiered scale modified from Chiu's method [31]. Liver pathologic scores were evaluated according to Eckhoff's reports [32].

### 2.4. Measurement of Serum Alanine Aminotransferase (ALT), Aspartate Aminotransferase (AST), Tumor Necrosis Factor (TNF)-*α*, and Interleukin (IL)-6 Levels

Collected blood was centrifuged (1000 g, 10 min, 4°C) and the serum was obtained. ALT and AST levels in the serum were measured according to the manufacturer's instructions supplied with the commercial assay kits (Jiancheng Bioengineering Biotechnology, Nanjing, China). TNF-*α* and IL-6 in the serum were determined using commercially available enzyme-linked immunosorbent assay kits according to the manufacturer's instructions (BOSTER Bio-Engineering Limited Company, Wuhan, China).

### 2.5. The Intestinal and Hepatic Antioxidant System Assay

The level of GSH and the activities of GSH-PX were analysed using commercial assay kits according to the manufacturer's instructions (Jiancheng Bioengineering Institute, Nanjing, China) and were expressed as mg GSH/g protein and U/g protein, respectively.

### 2.6. Semiquantitative Polymerase Chain Reaction (RT-PCR) for mRNA Analysis

The levels of mRNA expression of p66shc in the intestine and liver were determined by RT-PCR assays. Total RNA was extracted from mouse intestine and liver using TRIZOL reagent (Invitrogen, Carlsbad, CA, USA) according to the manufacturer's instructions. Reverse transcription into cDNA was performed using a TAKARA RNA PCR Kit (AMV) Ver. 3.0 (TAKARA, Dalian, China) for PCR analysis. The following primers were used: p66shc sense: 5′-ACTACCCTGTGTTCCTTCTTTC-3′, antisense: 5′-TCGGTGGATTCCTGAGATACTGT-3′; *β*-actin sense: 5′-AGAGGGAAATCGTGC-3′, antisense: 5′-CAATAGTGATGACCTGGCCGT-3′. All primers were synthesized by TAKARA. Five microliters of each RT-PCR were electrophoresed in a 1.5% agarose gel and stained with ethidium bromide. A BioSpectrum-410 multispectral imaging system with Chemi HR camera 410 (UVP, Upland, CA, USA) was used to analyze the intensity of the DNA bands. The intensity of each band was quantified by densitometry using a gel documentation and analysis system and normalized to values for *β*-actin.

### 2.7. Protein Extraction and Western Blotting

Tissues were homogenized in mammalian tissue protein extraction reagent (KeyGEN, Nanjing, China) supplemented with protease inhibitor and phosphatase inhibitor. For western immunoblotting, 25 to 60 *μ*g of protein was separated by 10–15% Bis-Tris protein gel (Bio-Rad, Hercules, CA, USA), transferred to polyvinylidene difluoride membranes (Millipore, Bedford, MA, USA), and blocked with 5% nonfat milk or 1% bovine serum albumin in tris-buffered saline tween-20 for 2 h at room temperature. The membranes were then incubated overnight with antibodies against p66shc, phospho-p66shc, foxo3a, manganese superoxide dismutase (MnSOD) (Abcam Ltd., Cambridge, UK); phospho-foxo3a (Beyotime Institute of Biotechnology, Hangzhou, China); NF-*κ*B (Santa Cruz Biotechnology, CA, USA); Bcl-xL (Proteintech group, Wuhan, China); cleaved-caspase-3 (Bioworld, Minneapolis, MN, USA); Histone H3.1 (Santa Cruz Biotechnology); or *β*-actin (Beyotime Institute of Biotechnology). After incubation with appropriate horseradish peroxidase-conjugated secondary antibodies, membranes were developed with ECL plus substrate (Beyotime Institute of Biotechnology). Images were documented with a BioSpectrum-410 multispectral imaging system with a Chemi HR camera 410 (UVP, Upland, CA, USA) and quantitated with the Gel-Pro Analyzer Version 4.0 (Media Cybernetics, Rockville, MD, USA).

### 2.8. Statistical Analysis

Data were expressed as mean ± standard deviation (SD). The statistical analysis was carried out using the SPSS16.0 statistical software package (SPSS Inc., Chicago, IL, USA). Statistical comparisons were analyzed by Kruskal-Wallis test for nonnormal distributions followed by Wilcoxon Rank Sum test with Bonferroni adjustments for multiple comparisons,or a one-way analysis of variance (ANOVA) followed by Student-Newman-Keuls (SNK) test for normal distributions. *P* value < 0.05 was considered statistically significant.

## 3. Results

### 3.1. PCA Pretreatment Decreased Histopathologic Damages of Intestine and Liver after Intestinal I/R

**Figure 1:**
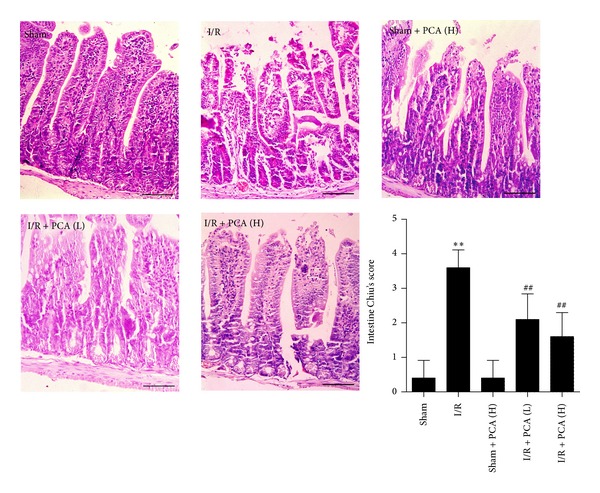
PCA pretreatment improved intestinal I/R-induced intestine histopathologic injury. Tissues were harvested at the end of reperfusion and were stained with hematoxylin eosin. Slides were examined under light microscopy at 200x magnification, bars = 50 *μ*m. Histologic injury scores in groups were quantified. Results are presented as the mean ± SD, *n* = 10. **P < 0.01 versus sham group; ^##^ *P* < 0.01 versus I/R group.

**Figure 2:**
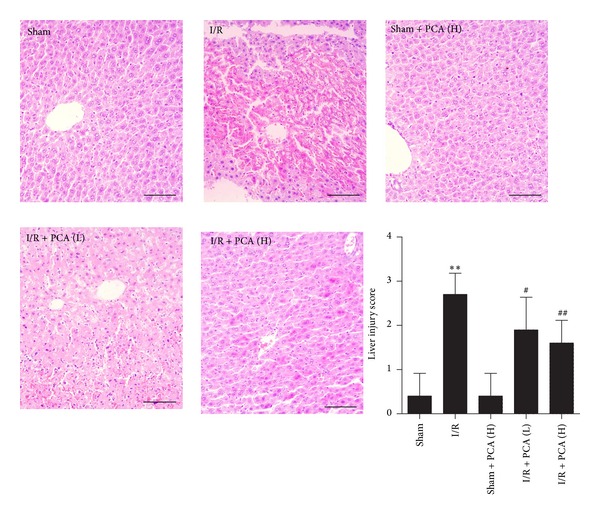
PCA pretreatment improved intestinal I/R-induced liver histopathologic injury. Tissues were harvested at the end of reperfusion and were stained with hematoxylin eosin. Slides were examined under light microscopy at 200x magnification, bars = 50 *μ*m. Histologic injury scores in groups were quantified. Results are presented as the mean ± SD, *n* = 10. **P < 0.01 versus sham group; ^#^ *P* < 0.05 versus I/R group; ^##^ *P* < 0.01 versus I/R group.

There were no obvious morphologic changes in the intestine or liver tissues in the sham and sham + PCA groups, whereas morphological changes from the I/R group showed significant histopathologic abnormalities compared with the sham group, including haemorrhage, severe villi irregularities, and edema in the intestine (Figure 1). Meanwhile, liver tissue was markedly damaged and showed cytoplasmic vacuolation, extensive nuclear pyknosis, and disintegration of hepatic cords (Figure 2). In the PCA pretreatment groups, the severity of histopathologic changes in the intestine and liver tissues was weaker than that in the I/R group (Figures 1 and 2). These results indicate that PCA confers protection against intestine and liver injury induced by intestinal I/R.

### 3.2. PCA Pretreatment Protected against Inflammation Damage and Liver Injury Induced by Intestinal I/R

**Figure 3:**
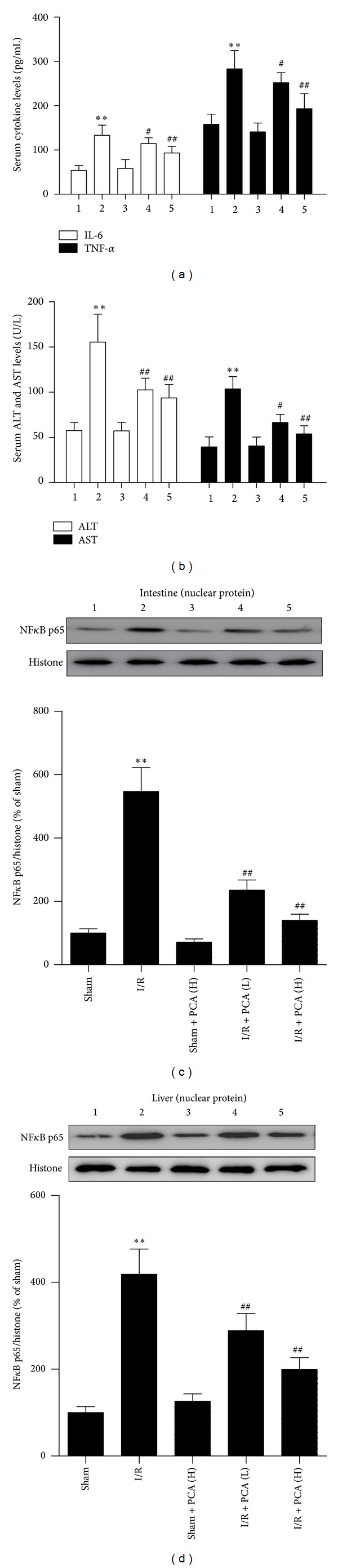
PCA pretreatment ameliorated intestinal I/R-induced systemic inflammation, hepatic dysfunction, and NF*κ*B activation. ((a) and (b)) Alterations of IL-6, TNF-*α*, ALT, and AST in the serum, *n* = 10. (c) Western blot analysis of nuclear NF*κ*B p65 in the intestine, *n* = 3. (d) Western blot analysis of nuclear NF*κ*B p65 in the liver, *n* = 3. Results are presented as the mean ± SD. **P < 0.01 versus sham group; ^#^ *P* < 0.05 versus I/R group; ^##^ *P* < 0.01 versus I/R group. 1 indicates Sham; 2, I/R; 3, Sham + PCA (H); 4, I/R + PCA (L); 5, I/R + PCA (H).

To determine whether intestinal I/R and PCA administration affect systemic inflammation, we measured TNF-*α* and IL-6 concentrations in serum. Compared with the sham group, TNF-*α* and IL-6 concentrations were significantly elevated in the serum of the I/R group. In contrast, our results showed that PCA pretreatment could prevent the increase in TNF-*α* and IL-6 production (Figure 3(a)). Serum ALT and AST levels were measured to assess liver function. As shown in Figure 3(b), after a 45-minute intestinal ischemia followed by a 90-minute reperfusion, ALT and AST levels in the serum were significantly increased compared with the sham group; after PCA pretreatment, a significant reduction in these parameters was observed in both groups compared to the I/R group. In addition, the nuclear expression of NF-*κ*B p65 subunit is critical for a wide range of processes such as inflammation [33]. We found that intestinal I/R-induced intestine and liver NF-*κ*B p65 subunit expression in the nuclear were inhibited by PCA pretreatment (Figures 3(c)-3(d)).

### 3.3. PCA Pretreatment Improved the Antioxidative Capacity of the Intestine and Liver after Intestinal I/R

The GSH system is generally considered to be a sensitive indicator of free radical scavenging ability. In this study, intestinal I/R caused a dramatic decrease in GSH and GSH-PX activities in the intestine and liver; however, PCA pretreatment improved GSH and GSH-PX activities in a dose-dependent manner (Table 1).

### 3.4. Expressions of p66shc in the Intestine and Liver Were Restrained by PCA Pretreatment after Intestinal I/R

**Figure 4:**
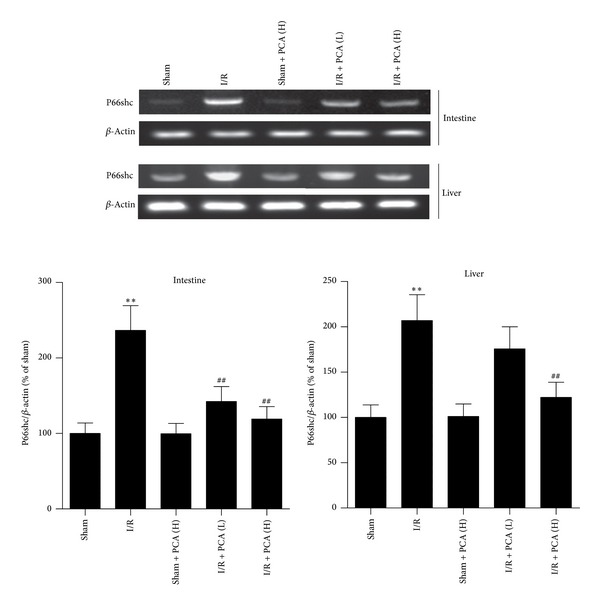
PCA pretreatment suppressed the mRNA expression of p66shc in the intestine and liver after intestinal I/R. Results are presented as the mean ± SD, *n* = 3. **P < 0.01 versus sham group; ^##^ *P* < 0.01 versus I/R group.

**Figure 5:**
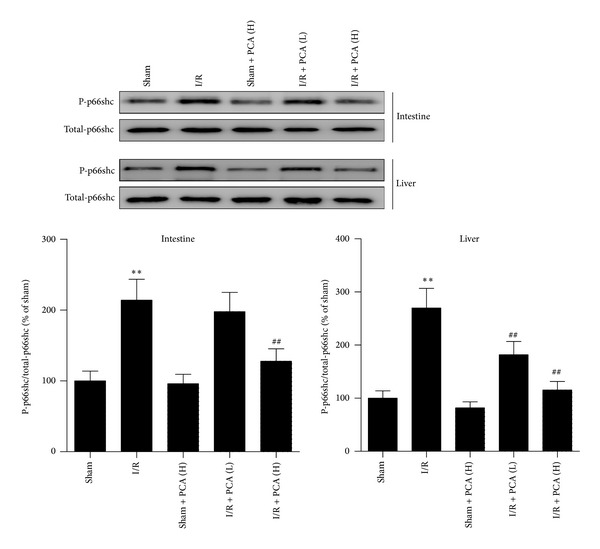
PCA pretreatment suppressed the phosphorylation of p66shc in the intestine and liver after intestinal I/R. Results are presented as the mean ± SD, *n* = 3. **P < 0.01 versus sham group; ^##^ *P* < 0.01 versus I/R group.

To determine whether p66shc plays a role in intestinal I/R injury and subsequent acute liver injury, as well as exploring the p66shc-regulation effect of PCA administration, RT-PCR and western blot analysis were carried out. Expression levels of p66shc mRNA were upregulated due to intestinal I/R in the intestine and liver, whereas PCA pretreatment resulted in a significant dose-dependent decrease in p66shc mRNA levels compared to I/R treatment. There were no differences in the sham + PCA groups compared with the sham group (Figure 4). Similarly, changes in the level of p66shc phosphorylation in the intestine and liver were significantly increased after intestinal I/R. PCA pretreatment could attenuate p66shc phosphorylation after intestinal I/R (Figure 5). These data show that intestine and liver p66shc mRNA expression and phosphorylation levels were elevated after intestinal I/R, while PCA pretreatment showed efficient alleviation of p66shc mRNA and phosphorylated protein levels, particularly in the 80 mg/kg PCA pretreatment group.

### 3.5. Effects of PCA Pretreatment on Oxidative-Stress Regulators of p66shc Signaling Pathway

**Figure 6:**
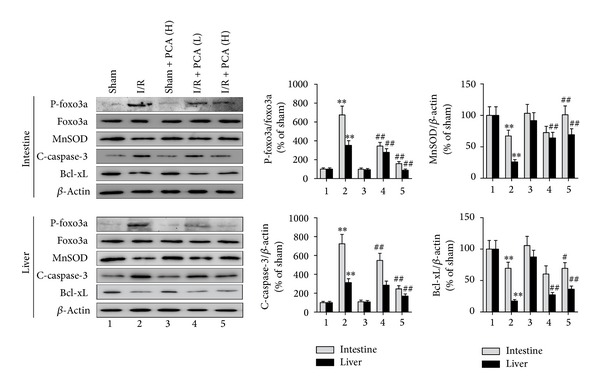
PCA pretreatment regulated the oxidative-stress regulators and apoptotic factors of p66shc pathway. Western blot analysis of phospho-foxo3a (p-foxo3a), foxo3a, MnSOD, cleaved-caspase-3 (C-caspase-3), and Bcl-xL in the intestine and liver. Results are presented as the mean ± SD, *n* = 3. **P < 0.01 versus sham group; ^#^ *P* < 0.05 versus I/R group; ^##^ *P* < 0.01 versus I/R group. 1 indicates Sham; 2, I/R; 3, Sham + PCA (H); 4, I/R + PCA (L); 5, I/R + PCA (H).

Foxo3a is a mammalian forkhead protein that tightly regulates the transcription of antioxidant genes such as MnSOD and catalase. Since previous reports indicated that foxo3a phosphorylation is associated with p66shc activation in response to oxidative stress [19], we further examined whether phosphorylation of foxo3a in the intestine and liver is involved in p66shc activation in response to intestinal I/R. As shown in Figure 6, expression levels of phospho-foxo3a were upregulated due to intestinal I/R in the intestine and liver, whereas PCA pretreatment resulted in a significant dose-dependent decrease in phospho-foxo3a levels compared to I/R treatment. However, there were no differences in the sham + PCA groups compared with the sham group. In contrast to phospho-foxo3a, expression of MnSOD in the intestine and liver after intestinal I/R following PCA pretreatment resulted in a significant dose-dependent increase in MnSOD levels compared to I/R exposure.

### 3.6. Effects of PCA on Apoptotic Factors in the p66shc Signaling Pathway

Several studies have shown that p66shc is a critical apoptotic component in a variety of pathophysiological processes. For example, high glucose levels induced greater caspase-3 activation in empty vector cells than in p66shc knockdown cells [34]. In the present study, intestinal I/R resulted in a significant increase in the intestine and liver cleaved-caspase-3 expression as compared to the sham-operated group, while PCA pretreatment prevented the intestinal I/R-induced increase of cleaved-caspase-3 expression. Moreover, expression of the antiapoptotic factor Bcl-xL in the intestine and liver was restrained because of intestinal I/R. However, PCA pretreatment markedly enhanced expression of Bcl-xL compared with the I/R group (Figure 6).

## 4. Discussion

Intestinal I/R, which is thought to be a triggering event in the development of local and distant organ dysfunction, remains a critical problem [35]. Multiple organ failure, particularly remote liver injury, is a recurrent complication of intestinal I/R injury and contributes to its high morbidity and mortality [9, 11–13]. In this study, we determined the effect of PCA on intestinal I/R injury and secondary severe liver injury mediated by p66shc suppression. Since PCA is commonly used as an antioxidative and stress-protective agent during multiple pathophysiological processes [26–28], we examined the protective effect of this agent during these processes. This study demonstrated that PCA pretreatment significantly attenuated intestinal I/R induced local intestine and remote liver morphological damage, oxidative stress response, systemic inflammation, and hepatic dysfunction, all of which resulted in improvement of the morbid state. Our results also demonstrated that PCA pretreatment had significant restraining effects on p66shc mRNA expression and protein phosphorylation after intestinal I/R in the intestine and liver, accompanied by p66shc-related oxidative stress regulators and apoptotic protein alteration.

A number of changes may occur in the intestinal mucosa and hepatocytes after acute intestinal I/R, leading to intestinal barrier dysfunction, bacterial translocation, systemic inflammation, and accompanying hypohepatia [8, 9, 11, 12]. In the present study, our results indicated that severe intestinal mucosal lesions and hepatic cell damage occurred after intestinal I/R, whereas PCA pretreatment significantly decreased these negative changes. This suggests that PCA can improve morphological alterations in the intestinal mucosa and hepatocytes in a murine model of intestinal I/R.

Several reports have indicated that impaired intestinal reperfusion after acute ischemia resulted in bacteria translocation into blood and other organs by damaging the intestinal barrier. The consequences of this process include inflammatory cytokine release, oxidative stress induction, and SIRS or MODS initiation [1, 4]. Most importantly, IL-6 and TNF-*α* are inflammatory cytokines and are commonly known to be elevated in the serum after intestinal I/R [15]. Once secondary liver injury is increased, ALT and AST are the clearest indicators of this process [14]. In this study, intestinal I/R resulted in the release of systemic inflammatory cytokines (IL-6, TNF-*α*) as well as elevation of liver enzymes (ALT, AST). Secondly, it has been demonstrated that activation of NF-*κ*B is a critical event for a wide range of processes such as inflammation [33]. We found that NF-*κ*B p65 was activated in the intestine and liver after intestinal I/R. Moreover, GSH and GSH-PX are generally used as critical indicators of antioxidative capacity [36]. Giovannini et al. [27] indicated that an impaired GSH antioxidant system promoted Caco-2 cells towards an apoptotic-prone phenotype by activating p66Shc expression. Similarly, our data showed that intestinal I/R resulted in reduction in the antioxidative capacity in the intestine and liver, manifested as GSH depletion and a reduction in GSH-PX activity. These alterations parallel the enhanced p66shc mRNA expression and phospho-p66shc upregulation. In this study, intestinal and liver p66shc mRNA expression and protein phosphorylation levels were suppressed by PCA, and the indicators described above were significantly improved. In summary, suppressing expression of p66shc by PCA pretreatment can improve the inflammatory response, liver dysfunction, and organ oxidative stress injury in the intestine and liver after intestinal I/R.

p66shc functions to regulate the level of intracellular ROS, cell apoptosis, and lifespan in mammals [19, 21, 37]. Its transduction properties in downstream signaling have recently attracted significant interest in stress-related research [17, 19]. Reports demonstrated that animals with a mutant p66shc gene induced stress resistance and prolonged lifespan [37]. Foxo3a belongs to the O subclass of the forkhead family and is thought to be a regulator of longevity and oxidative stress [38]. This gene encodes a variety of important antioxidant agents, such as MnSOD and catalase [19]. When oxidative stress occurs, ROS signaling activates p66shc through phosphorylation of a serine 36 residue. Phospho-p66shc can catalyze foxo3a phosphorylation andcytoplasm translocation, resulting in suppression of MnSOD expression [19]. Nemoto and Finkel demonstrated that dephosphorylation of foxo3a resulted in both an increase in ROS scavenging ability and oxidative stress resistance, whereas phosphorylation of foxo3a led to oxidative stress injury promotion. However, such injury induced by hydrogen peroxide is relieved in p66shc^−/−^ cells [19]. Additionally, AGE-induced phosphorylation of p66Shc selectively phosphorylated foxo3a and downregulated MnSOD in Human Embryonic Kidney 293 cells [39]. In the present study, p66shc was significantly phosphorylated in the intestine and liver after acute intestinal I/R, suggesting that p66shc phosphorylation is involved in the local intestine and remote liver injury induced by intestinal I/R. However, PCA pretreatment markedly reduced phosphorylation of p66shc in the liver and intestine. Additionally, our results revealed that intestinal I/R can lead to an increase in intestinal and hepatic foxo3a phosphorylation and downregulation of the transcription product MnSOD, whereas PCA pretreatment reversed these effects. Our results indicate that phosphorylation of foxo3a may be mediated in part by intestinal I/R-induced p66shc phosphorylation in the intestine and liver. Because phosphorylation of p66shc was suppressed, PCA suppressed foxo3a phosphorylation and enhanced MnSOD expression.

Increasing data support a viewpoint that varying degrees of multiple organ apoptosis occur during the pathogenesis of intestinal I/R [9, 10, 40]. Horie et al. [14] found that rats exposed to intestinal I/R caused an increase in apoptotic hepatocytes. Previous reports demonstrated that cleaved-caspase-3 and Bcl-xL were sensitive indicators of p66shc-induced apoptosis [34, 41]. Therefore, we detected cleaved-caspase-3 and Bcl-xL to reflect the proapoptosis and antiapoptosis levels of the intestine and liver in the present study. These results showed that intestinal I/R-induced intestine and liver p66shc phosphorylation were accompanied by cleaved-caspase-3 upregulation and Bcl-xL downregulation, whereas PCA pretreatment significantly reversed this trend. Consistent with previous reports [34, 41], we inferred that intestinal I/R can result in cell apoptosis in the intestine and liver, and such damages were characterized by p66shc phosphorylation-induced caspase-3 activation. In contrast, PCA pretreatment may protect intestinal epithelial cells and hepatocyte from apoptosis induced by intestinal I/R through upregulation of the antiapoptotic gene Bcl-xL following p66shc suppression.

In summary, we demonstrated that PCA had significant protective effects against local intestine and remote liver injury induced by intestinal I/R in mice, and that its protective effects appeared to involve inhibition of p66shc. Furthermore, such protective effects may be mediated in part by foxo3a activation, MnSOD upregulation, and enhanced Bcl-xL expression involved in the p66shc signaling pathway. Accordingly, PCA may be a potential candidate for improving intestinal I/R injury and secondary liver damage. Additionally, manipulation of p66shc phosphorylation may offer an attractive therapeutic means for alleviating multiple organ injury in intestinal I/R patients.

## Figures and Tables

**Table 1:** PCA pretreatment ameliorated intestinal I/R induced GSH system dysfunction in the intestine and liver.

Group	Intestine GSH (mg/g protein)	Intestine GSH-PX (U/g protein)	Liver GSH (mg/g protein)	Liver GSH-PX (U/g protein)
Sham	3.71 ± 0.48	87.73 ± 10.51	2.45 ± 0.17	424.31 ± 22.06
I/R	2.25 ± 0.55**	43.54 ± 8.61**	1.54 ± 0.09**	314.16 ± 26.58**
Sham + PCA (H)	3.81 ± 0.56	94.38 ± 19.45	2.29 ± 0.23	418.56 ± 15.72
I/R + PCA (L)	2.75 ± 0.53	69.85 ± 10.58^##^	1.76 ± 0.08	357.01 ± 8.51^##^
I/R + PCA (H)	3.23 ± 0.16^##^	72.40 ± 11.03^##^	2.07 ± 0.17^##^	379.42 ± 4.39^##^

Results are presented as the mean ± SD, *n* = 10. **P < 0.01 versus sham; ^##^ *P* < 0.01 versus I/R.
